# That's So Last Season: Unraveling the Genomic Consequences of Fur Farming in Arctic Foxes (*Vulpes lagopus*)

**DOI:** 10.1111/mec.70166

**Published:** 2025-11-13

**Authors:** Christopher A. Cockerill, J. Camilo Chacón‐Duque, Nora Bergfeldt, Johanna von Seth, Gabriella Björklund, Malin Hasselgren, Johan Wallén, Anders Angerbjörn, Eva Fuglei, Ester Rut Unnsteinsdottir, Paula White, Gustaf Samelius, Ray Alisauskas, Dominique Berteaux, Øystein Flagstad, Arild Landa, Nina E. Eide, Remi‐André Olsen, Ignas Bunikis, Snæbjörn Pálsson, Kristinn Pétur Magnússon, Love Dalén, Karin Norén

**Affiliations:** ^1^ Department of Zoology Stockholm University Stockholm Sweden; ^2^ Centre for Palaeogenetics Stockholm Sweden; ^3^ Department of Bioinformatics and Genetics Swedish Museum of Natural History Stockholm Sweden; ^4^ Norwegian Polar Institute, Framsenteret Tromsø Norway; ^5^ Icelandic Institute of Natural History Garðabær Iceland; ^6^ Institute for the Environment and Sustainability University of California Los Angeles California USA; ^7^ Snow Leopard Trust Seattle Washington USA; ^8^ Department of Biology University of Saskatchewan Saskatoon Saskatchewan Canada; ^9^ Wildlife Research Division, Environment and Climate Change Prairie and Northern Wildlife Research Centre Saskatoon Saskatchewan Canada; ^10^ Canada Research Chair on Northern Biodiversity Université du Québec à Rimouski Rimouski Quebec Canada; ^11^ Centre for Northern Studies Université du Québec à Rimouski Rimouski Quebec Canada; ^12^ Norwegian Institute for Nature Research Trondheim Norway; ^13^ Department of Biochemistry and Biophysics, Science for Life Laboratory Stockholm University Solna Sweden; ^14^ Department of Immunology, Genetics and Pathology, Uppsala Genome Center, National Genomics Infrastructure Hosted by SciLifeLab Uppsala University Uppsala Sweden; ^15^ Department of Life and Environmental Sciences University of Iceland Reykjavik Iceland; ^16^ Faculty of Natural Resource Sciences University of Akureyri Akureyri Iceland

**Keywords:** arctic fox, demographic history, domestication, whole‐genome sequencing

## Abstract

Humans have relied on animal fur for centuries, yet fur farming only began recently during the mid‐19th Century. Little is known about this incipient domestication or the genomic processes involved. Domestication may involve founder effects, population bottlenecks and low population size, which, when combined with intense artificial selection, lead to inbreeding, a limited gene pool and reduced fitness. The arctic fox ( *Vulpes lagopus* ) has been farmed intensively since the early 1900s and has been artificially selected for economic phenotypes. We investigated the origin of these lineages and the genomic consequences of intensive farming by comparing the genomes of farmed and wild arctic foxes from across their range. Our research indicates recent inbreeding through long Runs of Homozygosity and reduced genomic variation in farmed foxes relative to their respective wild populations. We identified a coastal ecotype origin for all Fennoscandian farmed arctic foxes, aligning them phylogenetically with the wild Icelandic population, a geographically isolated and phenotypically distinct coastal lineage. The depleted genome‐wide heterozygosity and increased recent inbreeding in farmed fox lineages is consistent with a heavy consequence of domestication, shedding light on the demographic history and genomic consequences of human manipulation. We highlight the need for increased genomic investigations into fur farm populations to understand the incipient domestication process and uncover the cost of intense farming. The genomic consequences of domestication must be considered in the management of fur farms, with actionable steps needed to prevent descendants of escaped farmed foxes from polluting the gene pool in the wild through introgression.

**Abbreviations**
BAM: Binary Alignment/Map file
BP: before present
*F*ROH: genomic inbreeding coefficent
F*ST*
: fixation index
Ne: effective population size
PCA: principal component analysis
ROH: runs of homozygosity
SFS: site frequency spectrum
SNP: single nucleotide polymorphism
VCF: variant call format

## 1 Introductions

Animal pelts have been harvested as a necessary survival tactic that allowed humans to disperse across the globe and tolerate the more inhospitable reaches of the north (Gilligan 2010). In particular, pelts of cold‐adapted species with specialised layers of fur to minimise heat loss, were favoured by humans as a source of insulation (Stenton 1991). Long after the hunter‐gatherer days, the demand for furs increased, and by the 20th century furs became a fashion statement, indicating wealth and status. However, wild animal trapping is challenging and the yield is unpredictable. Thus, fur suppliers shifted to fur farming, harvesting captive animals akin to typical livestock farming. Animals have been used for human benefit for thousands of years, and artificial selection, or “domestication” has been ubiquitous for the past 15,000 years (Frantz et al. 2020). Artificial selection, (i.e., breeding individual animals of desired phenotype over several generations), is widespread across the globe, and many old, well‐known breeds have been selected for over thousands of years for different purposes, including bovines (∼12,000 years Before Present; BP; Zhang et al. 2020), pigs (∼10,000 years BP; Groenen 2016), chickens (∼8000 years BP; Lawal and Hanotte 2021), horses (∼4000 years BP; Orlando 2020) and donkeys (∼7000 years BP; Todd et al. 2022).

Such domesticated breeds have long diverged from their wild counterparts, having been selected for desirable and economic traits like increased body size, increased milk yield and fecundity. Domestication is a difficult concept to define, but it can be viewed as a form of evolution shaped by human influence rather than natural selection (Driscoll et al. 2009). Domestication is, however, a process that may favor production over individual fitness, although the goal outcome of this form of selection depends on the motivations of individual farmers. The fur farming industry harvests fur from captive domesticated animals, usually from individuals selectively bred for excess skin, thick fur and fecundity to maximize fur yield and economic return. Beginning with attempts to farm mink during the American Civil War (1861–65; Obbard et al. 1987), fur farming became widespread, reaching its peak in the 1980s. Although several countries have banned or reduced fur farming over the last two decades (Fur Farming Prohibition Act, 2000; Arney 2022), the industry still thrives in various countries across the globe such as Finland, Canada, China, Iceland, Japan, Russia and the USA (Warwick et al. 2023) and involves a range of species (i.e., mink, foxes, rabbits). Fur farming is a recent practice relative to ancient domesticates such as dogs and cattle, yet animals farmed for their fur can be loosely defined as being in a semi‐domesticated state akin to the early stages of domestication. However, where tameness was often a key priority during traditional domestication, fur farming typically focused more on morphological phenotype (Thirstrup et al. 2019).

Animal domestication has become a highly studied and controversial topic, from the origin, timing and process of the most familiar and iconic domesticates (i.e., livestock; Vigne 2011; and dog domestication; Galibert et al. 2011), to the debated ubiquity of the domestication syndrome (i.e., categorizing a suite of traits that define domesticated species; Lord et al. 2020). While semantic disputes may produce interesting lines of research and debate, it is also necessary to focus our attention on understanding the consequences of domestication, such as the geographic origin of selected stock, potential founder effects and uninformed breeding strategies, which may have been overlooked during early domestication attempts with limited knowledge of animal husbandry, or the negative impacts of inbreeding and reduced genetic variation. Several studies have sought to unravel the origin of domesticated lineages (Verdugo et al. 2019; Wang et al. 2020; Lavretsky et al. 2023; Champagnon et al. 2023; Rando et al. 2024), which can be challenging since historical accounts of animal handling can be difficult to access, obscuring evidence of intentional inbreeding and bottlenecks that gave rise to most economic, marketable phenotypes (Kristensen et al. 2015). This is important knowledge given the ongoing risk of domesticated lineages interbreeding with several wild populations (e.g., extensive global introgression of domestic ducks into wild duck populations; Lavretsky et al. 2023; domestic dogs into wolves; Pilot et al. 2021; domestic cats into European wildcats; Hertwig et al. 2009; Nussberger et al. 2014; Senn et al. 2019; domestic pigs into wild boar; Frantz et al. 2013; Dzialuk et al. 2018).

Over the past decades, several countries have implemented legislation to prevent cruelty through extreme selection; however, they prove difficult to enforce (Olsson et al. 2006). As a result, in practice, the domestication process is often approached haphazardly and may involve using founders from several source populations or from a single origin. The genetic implications of these different scenarios would have contrasting outcomes; for example, unrelated source populations may have intrinsic genetic incompatibilities, whereas sourcing individuals from a single origin would mean less genetic variation to buffer against potential detrimental outcomes (e.g., inbreeding depression). These contrasting origins would influence subsequent microevolutionary changes, with the persistence of incompatible genotypes, and accelerated genetic drift following a founder effect, respectively. Over time, wild, unrelated individuals may be added to the captive population to provide new genetic variation or preferred phenotypes for further selection. However, processes including relaxed selection (i.e., the removal of selection in an artificial environment; Christie et al. 2012), amplified genetic drift (i.e., the stochastic loss and fixation of alleles; Moyers et al. 2018), inbreeding (i.e., mating between close relatives) and inbreeding depression (i.e., decreased individual fitness through inbreeding; Bosse et al. 2019) and a heightened mutational load (i.e., increased deleterious mutations segregating in the population; Ollivier 1980; Mignon‐Grasteau et al. 2005; Lu et al. 2006; Bosse et al. 2019) can impact the viability of captive populations. Indeed, an increased mutational load in domesticated species has been coined the “cost of domestication” hypothesis (Lu et al. 2006), which suggests that bottlenecks and reduced efficiency of purifying selection in domestic species lead to an accumulation of deleterious mutations.

The process of domestication has been investigated using a variety of traditional genetic tools (Bruford et al. 2003; Larson and Burger 2013). With the advent of whole‐genome sequencing, levels of inbreeding and mutational load have subsequently been identified in several species such as dogs (Cruz et al. 2008; Marsden et al. 2016), horses (Schubert et al. 2014), cattle (Zhang, Calus, et al. 2015; Zhang, Guldbrandtsen, et al. 2015), livestock (Charlier et al. 2008, 2016) and pigs (Derks et al. 2019). Efforts have been made within the livestock industry to incorporate genomic tools to maintain genetic variation and identify deleterious variants (Windig et al. 2004; Windig and Doekes 2018; Charlier et al. 2008, 2016; Derks et al. 2019). However, while knowledge regarding the early stage of domestication remains limited, the incipient stages of domestication that are represented by fur‐farmed animals are important for understanding which micro‐evolutionary processes shape the domestic species.

The arctic fox ( *Vulpes lagopus* ) is a species for which populations exhibit vastly contrasting demographic histories across its range. Because arctic fox fur provided a crucial source of insulation for humans during the expansion of human populations into the north, the species has experienced varying levels of hunting pressure across its circumpolar distribution. Arctic foxes are recognised as two evolutionary distinct ecotypes with unique life histories: the generalist coastal fox of the Alaskan coast and islands, western Greenland, Iceland, and Svalbard (stable, inter‐annual diet of marine carcasses, sea birds and eggs) and the specialist lemming fox of inland Alaska, eastern Greenland, the Fennoscandian Peninsula, Siberia, Alaska and Canada (3–5‐year fluctuating cycles driven by rodent species; Braestrup 1941). To supply the fashion industry, arctic fox farms were established in the 20th century in North America (Konnerup‐Madsen and Hansen 1980; Nes et al. 1987). The first farms were established on islands in the early 1900s, which were populated with “blue fox” stock (Lucas 1900; Ashbrook and Walker 1925). The individuals were named according to their coat morph, with which there are three variants observed in the wild: white, sand and blue, the last being the most common in the coastal ecotype. Thereafter, arctic fox farms were established in several countries (Norway, 1913; Nes et al. 1987; Finland, 1924; Frafjord 1993; Mustonen et al. 2006), and the lineages of these farms may originate from Alaska, Greenland, Svalbard, Jan Mayen and Icelandic populations (Nes et al. 1987; Johansson 1950; Mustonen et al. 2006; Norén et al. 2005). Subsequently, farmed arctic foxes were selectively bred to maximise traits such as excess skin, fur quality, fecundity and body size. However, inbreeding depression, which reduces litter size and juvenile survival (Wierzbicki et al. 2004), can counteract these breeding goals (Nordrum 1994).

Following escapes and releases of farmed foxes into the wild, hybridization between farmed and wild foxes has been detected (Frafjord, 1985 in Linnell et al. 1999; Hersteinsson et al. 1989; Hersteinsson 2004). In Fennoscandia, entire sub‐populations of farmed foxes have been documented over the past century, in Østerdalen (Eide and Hamlander 1933) and Hardangervidda (Norén et al. 2009). According to mitochondrial‐based population genetics analyses, Fennoscandian farmed foxes (i.e., Norway, Sweden and Finland) can be traced back maternally to Alaska, Greenland and Svalbard stock (Norén et al. 2005; Dalén et al. 2005). Consequently, introgression into the wild populations in Fennoscandia could lead to outbreeding depression (i.e., reduced fitness caused by the breakdown of locally adapted gene complexes), or introduce new deleterious variation.

The aims of this study were to leverage high‐coverage modern and historical whole genomes from captive‐farm and wild arctic fox populations across the circumpolar range to investigate the phylogenetic placement of farmed fox lineages with a specific goal to determine the ecotype origin of the domestic lineages. Further, we also aim to reconstruct demographic histories to identify bottlenecks that occurred during the incipient domestication process, and to determine resulting genome‐wide heterozygosity and inbreeding levels.

We hypothesize that given historical accounts and existing genetic evidence, the farmed arctic foxes should (1) show signatures of a mostly coastal heritage, (2) show signs of recent bottleneck events, with increased inbreeding coinciding with the establishment of respective farms and (3) show reduced genomic diversity relative to wild populations.

## 2 Methods

### 2.1 Study Populations

**FIGURE 1:**
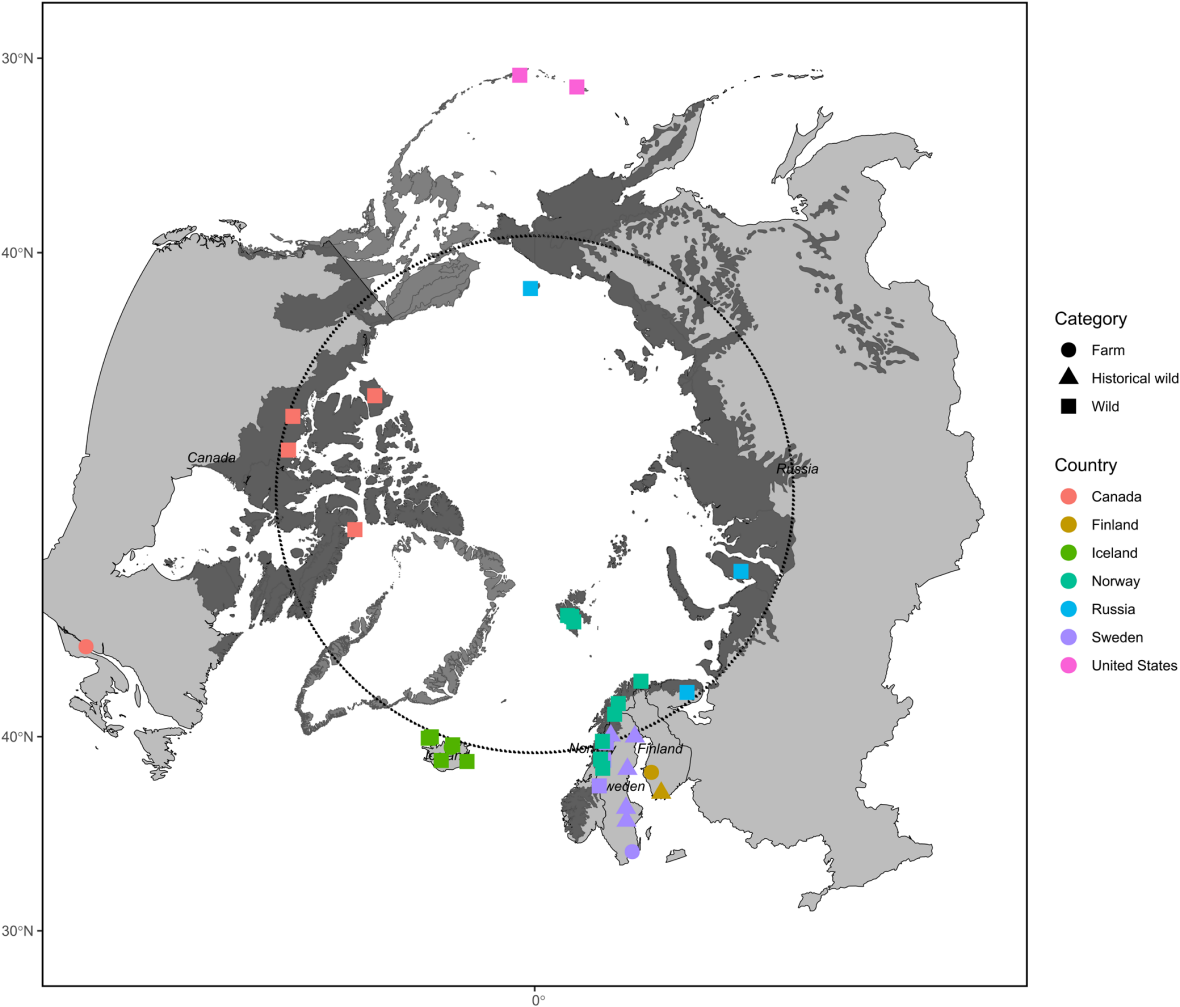
The circumpolar Arctic tundra, study sample areas are shown in the United States, Canada, Iceland, Norway, Sweden, Finland and Russia. Wild and farmed samples are indicated by squares and circles, respectively. Historical samples are represented by triangles.

The study system spans the circumpolar Arctic region, encompassing the North American continent (United States and Canada) and Eurasia (Iceland, Norway, Sweden, Finland and Russia; Table 1 and Figure 1). In North America, samples were sourced from Alaska (wild) and the Aleutian Islands archipelago in the United States (farm: Shemya Island, Near Islands and Tanaga Island, western Andreanof Islands), Canada (wild: Banks Island, Bylot Island, Karrak Lake and Bathurst Inlet; farm: Uncertain—either Nova Scotia or Newfoundland). The samples from the Aleutian Islands represent non‐native arctic fox “colonies”, which were introduced to the islands, potentially representing the first farm populations. In Eurasia, samples were sourced from Iceland (wild: Jökuldalur, Eyjafjörður, Skeiða og Gnúpverjahreppur, Súðavík and Vesturland; farm: Möðruvellir, Hörgárdalur), Sweden (farm: Blekinge), Norway (wild: Svalbard; farm: Gol and Ål, Hallingdal) and Finland (wild historical: Ahvenanmaa). Three Icelandic samples were labelled as putative farmed foxes, as they were previously identified in situ based on physical attributes of farmed foxes. Previously published sequencing data were utilised from Hasselgren et al. (2021; Helagsfjället, Sweden), Cockerill et al. (2022; Vindelfjällen and Arjeplog, Sweden; Saltfjellet, Reisa nord, Varangerhalvøya and Øvre Dividal, Norway; Yamal, Taymyr, Indigirka, Faddeyevsky Island, Wrangel Island and Kola, Russia) and von Seth et al. (2025; wild historical: Kvikkjokk, Södertälje, Södermanland, Härnösand, Runmarö, and Uppland, Sweden). To represent the Finnish farmed fox lineage, short read Illumina sequencing data of a blue fox was downloaded from (Peng et al. 2021).

**TABLE 1:** Summary of population, country, type (heritage), material, number of individuals used for whole genome analyses. Samples sequenced for this study are in parentheses.

Population	Country	Type	Material	*n*	Citation
Fennoscandia	Norway	Wild modern	Skin	7	Cockerill et al. 2022; von Seth et al. 2025
		Farm modern	Claws	7 (7)	—
	Sweden	Wild modern	Skin	25	Hasselgren et al. 2021; Cockerill et al. 2022
		Farm modern	Skin	4 (4)	—
		Wild historical	Bone	4	von Seth et al. 2025
	Finland	Wild historical	Bone	1	von Seth et al. 2025
		Farm modern	Blood	1	Peng et al. 2021
	Russia (Kola)	Wild modern	Skin	1	Cockerill et al. 2022
Siberia	Russia	Wild modern	Skin, Muscle	3	Cockerill et al. 2022
Svalbard	Norway	Wild modern	Muscle	3 (3)	—
North America	United States	Farm modern	Tissue	2 (2)	—
	Canada	Wild modern	Skin	4 (4)	—
		Farm modern	Skin	1 (1)	—
Iceland	Iceland	Wild modern	Muscle	5 (5)	—
		Farm modern	Skin, Fur	3 (3)	—
Total				71 (29)	

A dataset of 71 re‐sequenced whole genomes was collected. For this study, we generated sequencing data for 29 samples (Table 1). Published sequencing data from Sweden, Norway and Russia were available from previous research and the data from (Peng et al. 2021) was downloaded from the NCBI database.

Samples were collected between 1989 and 2022 using a broad range of sampling strategies. The wild samples from Sweden (Helagsfjället, Vindelfjällen, Arjeplog) and Canada (Banks Island, Bylot Island, Karrak Lake and Bathurst Inlet) were collected as ear tissue biopsies (i.e., byproducts of ear‐tagging for monitoring purposes); wild samples from the United States were collected as tissue samples from carcasses salvaged during monitoring of Shemya Island (i.e., road kill) and from carcasses acquired from an eradication attempt of foxes on Tanaga Island conducted by The United States Fish and Wildlife Service (USFWS). The wild samples from Iceland and Svalbard were collected from carcasses acquired from fox trappers. The farmed fox samples were collected as tissue (Sweden, Norway, Iceland and North America), claw clippings (Norway) and fur (Iceland). Tissue samples were placed in 99% ethanol and stored in −20°C, or were frozen directly until extractions, except for the Icelandic samples which were stored in a desiccated state.

Ear‐tagging in Sweden was approved with ethical permits from the Swedish Board of Agriculture and additional allowances from the Swedish Environmental Protection Agency (A130‐07, A131‐07, A36‐11, A18‐14, A19‐14, 412–7884‐07 NV, NV‐01959‐14). Ear‐tagging in Karrak Lake was done in compliance with Canadian Council of Animal Care guidelines under the University of Saskatchewan Committee of Animal Care and Supply Protocol number 19990029.

### 2.2 De‐Novo Assembly

We generated an arctic fox genome assembly using a combination of PacBio long‐read sequencing and Hi‐C scaffolding. A Dovetail Hi‐C sequencing library was prepared according to the protocol included by the manufacturer (“Hi‐C Kit Manual v.1.03 4‐13‐18”) of the prep kit. Frozen muscle tissue from one male and one female, Swedish wild individual (not included in downstream analyses), born in 2015 and 2013, respectively, was ground in liquid nitrogen using a mortar and pestle and a portion (33.1 mg) of the ground material was used as input for crosslinking by formaldehyde (i.e., the first step of the protocol).

For the PacBio sequencing, HiFi SMRTbell libraries were constructed from the high molecular weight DNA extraction of the same male Swedish individual born in 2015, as described in “Procedure & Checklist—Preparing HiFi SMRTbell Libraries” (PN 101‐853‐100 Version 03 January 2020) using the SMRTbell Express Template Prep Kit 2.0 (PacBio). 15 μg of DNA was used for shearing on the Megaruptor 2 DNA Shearing System to 20 kb followed by SMRTbell library construction. Size selection was performed using the SageELF system (SageScience). The fractions with suitable fragment lengths obtained during size selection, went on to sequencing. Primer annealing and polymerase binding were performed using the Sequel II binding kit 2.0 and Sequencing Primer v2. Finally, four Sequel SMRT Cells 8 M were sequenced on Sequel II using Sequel II Sequencing Plate 2.0, On‐Plate Loading Concentration of 90 pM, movie time 30 h and pre‐extension time 2 h.

The Hi‐C library was sequenced in a single flowcell lane of an Illumina HiSeqX instrument (HiSeq Control Software HD 3.5.0.7/RTA 2.7.7) using a 2 × 151 setup and ‘HiSeq X SBS’ chemistry. After demultiplexing by bcl2fastq (v2.19.1.403), the final sequencing yield was 417.6 M clusters.

PacBio long read data was assembled using IPA HiFi Genome Assembler v1.5.0 (PacBio, https://github.com/PacificBiosciences/pbipa). HiC read mapping to the contig assembly and pre‐processing was performed using Juicer v.1.6 (Durand et al. 2016) with the command‐line parameter “‐s none” to disregard restriction sites. Draft scaffolding was generated by running 3D‐DNA (v180922, git‐SHA: 529ccf4) with the parameter “‐r 0” for minimal error correction (Dudchenko et al. 2016). The scaffolded assembly was manually curated in Juicebox Assembly Tools (v1.11.08; Dudchenko et al. 2018).

### 2.3 Genome Sequence Data Processing

Raw sequencing data were processed using the GenErode bioinformatics pipeline v0.5.1 (Kutschera et al. 2022). First, adapters were trimmed from forward and reverse reads using fastp v0.22.0 (Chen et al. 2018). Reads were then aligned to the constructed genome assembly generated using PacBio and Hi‐C reads (PRJEB71153). For mapping, we used the BWA mem algorithm (v0.7.17) for modern data and BWA aln for historical data. PCR duplicates were sorted and removed using SAMtools v1.12, and reads were realigned around indels. Repeat regions, CpG sites, scaffolds shorter than 25,000 bp and scaffolds linked to the X chromosome were masked using BEDtools v2.29.2 (Quinlan and Hall 2010). Moreover, bases with mapping quality (MQ) < 30 were filtered out. The average depth of coverage was estimated with zero‐coverage sites excluded (Kutschera et al. 2022; Li et al. 2009; McKenna et al. 2010; Smit et al. 2023, 2008). To minimise biases due to uneven coverage while maintaining sufficient data for downstream analyses, we subsampled BAM files with > 8× coverage to 8× and retained only those with an average depth between 5× and 8×, resulting in a final dataset of *n* = 71. For generating single nucleotide polymorphisms (SNPs), variants were called on the filtered BAM files greater than 7× using bcftools mpileup and bcftools v1.8 with a minimum depth of approximately ∼1/3 of the average coverage (i.e., 2.7×) and a maximum depth greater than twice the average coverage (i.e., 16×). Variants were further filtered by base quality (QV ≥ 30) and by excluding those within 5 bp of indels. After merging all individual VCF files, we obtained 12,341,480 high‐quality SNPs. SNPs with heterozygous allele frequencies outside the allelic balance range (i.e., < 0.2 and > 0.8) were removed to avoid biases caused by contamination, mapping, or sequencing errors. Additionally, variants called in fewer than 95% of individuals were excluded, resulting in a final dataset of 1,201,361 high‐quality, genotyped SNPs.

We estimated the genotype likelihoods using ANGSD (Korneliussen et al. 2014) with the filtered BAM files as input according to the GATK model (−GL 2), inferred major and minor alleles (−doMajorMinor 1), and limited the output to only biallelic sites (only polymorphic sites: −SNP_pval 1 × 10^−6^, no triallelic sites: −skipTriallelic 1). We only kept sites that were present in at least 94% of all individuals (−minInd 68; i.e., 68 individuals), generating a dataset for 53 wild and 18 farmed foxes. All downstream analyses using this dataset only included regions that had mapping scores > 30 within the 24 longest scaffolds, each above 25 k base pairs long.

### 2.4 Population Structure, Differentiation and Demographic History

To characterise population structure, we used PLINK v.1.9 (Chang et al. 2015) and PCAngsd (Meisner and Albrechtsen 2018) to produce the covariance matrix to perform a Principal Component Analysis (PCA) independently, using the SNP data and genotype likelihoods. This allowed us to compare PCAs generated based on variant calling and GL to assess the presence of artefacts due to missingness in the datasets. We used NGSadmix (Skotte et al. 2013) to calculate admixture proportions of individuals using GL, running ancestry clustering of K = 1 through K = 10 until model convergence, until the top 5 maximum likelihood runs were consistent. To identify the best K value, we used CLUMPAK (Kopelman et al. 2015) based on five replicates for each K according to the method of (Evanno et al. 2005). For further evaluation, we calculated the residual correlations using EvalAdmix (Garcia‐Erill and Albrechtsen 2020) to assess whether the data violated the assumptions of the model.

To quantify genomic differentiation between populations, we estimated pairwise F_*ST*_ values between all population pairs in ANGSD (Korneliussen et al. 2014). We used three individuals from each population to allow for unbiased comparisons. First, we calculated the unfolded site allele frequency (−doSAF 1, parameters: −GL 1; −minMapQ 30; −minQ 30; −minInd 3; −uniqueOnly 1; −only_proper_pairs; −remove_bads 1; −do_Maf 1; −doCounts 1, −doGLF 2; −doMajorMinor 1; −C 50). We then calculated the joint Site Frequency Spectrum (SFS) for each pair using the realSFS command. Next, we calculated the global estimates for F_*ST*_ values between populations using the realSFS fst stats command and we report the weighted F_*ST*_ value (Weir and Cockerham 1984).

To infer recent demographic history of the farmed arctic fox (< 10,000 years BP), we used SMC++ v.1.15.2 (Terhorst et al. 2017) and GoNe (Santiago et al. 2020) on Fennoscandian farmed arctic foxes. We chose only modern genomes that were assigned 100% to the same population in the admixture analyses up to K = 10, clustered within the same clade in the phylogeny and had the shortest branch lengths to minimise the effects of population structure and genetic drift (*n* = 12). SMC++ is an approach that harnesses Sequential Markov Coalescence (SMC) to estimate effective population size (*Ne*). We used a mutation rate of 1 × 10^−8^ per site^−1^ per year^−1^ and a generation time of 1.5 years consistent with the average maternal age in an arctic fox fur farm (Kempe and Strandén 2016). Since the mutation rate of the arctic fox is unknown, we tested three different mutation rates according to *Canis lupus* estimates commonly used in the literature (4.5 × 10^−9^, 1 × 10^−8^, 2.5 × 10^−8^; Koch et al. 2019). We used a cross‐validation approach with –em‐iterations 5000, −thinning 1070, −knots 11 and –regularisation‐penalty 6.

To provide more robust estimates of demography over the last 400 years, we used GoNe to estimate changes in N_*eLD*_ (Santiago et al. 2020), the geometric mean over 40 independent runs from the observed spectrum of Linkage Disequilibrium (LD). We only included the 24 longest autosomal scaffolds and ran the analysis using PHASE = 2; cMMb = 1; DIST = 1; NGEN = 2000; NBIN = 400; MAF = 0.0; ZERO = 1; maxNCHROM = 85; maxNSP = 50,000; hc = 0.05; REPS = 40; threads = 99.

### 2.5 Phylogenetic Analyses

To investigate the origin of farmed arctic foxes, we generated a nuclear maximum likelihood phylogeny using the red fox ( *Vulpes vulpes* ) as the outgroup using IQ‐Tree v2.2.0 (Nguyen et al. 2015; Minh et al. 2020). We performed random allele sampling on BAM files above 6× using ANGSD (−dohaplocall 1, parameters: ‐uniqueOnly 1, −remove_bads 1, −minMapQ 30 −minQ 30, −doCounts 1, −minMinor 1). The haploid files were sub‐sampled for every 100th line using awk and converted into FASTA format using a custom python script. ModelFinder (Kalyaanamoorthy et al. 2017), implemented by IQ‐Tree, determined *TVMe* + *ASC* + *G*4 as the best‐fitting model according to the Bayesian Information Criterion (BIC). Branch support was inferred using ultrafast bootstrap (UFboot; Hoang et al. 2018).

### 2.6 Genome‐Wide Heterozygosity and Inbreeding

Using the filtered BAM files, we estimated genome‐wide heterozygosity based on the individual mutation rate (*θ*) using mlrho v2.7. This method follows the infinite sites model, where *θ* estimates the number of heterozygous sites per 1000 bp.

We used Runs of Homozygosity (ROH) identified using the PLINK v1.9 sliding‐window method to estimate inbreeding coefficients (F_*ROH*_). We tested parameters according to published literature (Hasselgren et al. 2021; Cockerill et al. 2022), and found that restricting the data to sites found in 100% of individuals reduced the number of SNPs. This is likely due to the degraded nature of the various sample materials (i.e., claw clippings, desiccated tissue), therefore, as described above, we filtered the VCF to allow 5% missingness. Including missingness up to 10% is routine for historical studies using the Generode pipeline and robust results have been demonstrated in various empirical studies based on modern samples (von Seth et al. 2022; Whitla et al. 2024). To ensure this did not affect downstream results, we compared ROH lengths of mutual data with those obtained using 0% missingness from (Cockerill et al. 2022), demonstrating near identical results (Figure S1). In addition, we fine‐tuned three specific parameters: allow at most 1 heterozygous call per window to infer homozygous window (homozyg‐window‐het 1), sliding window size 100 SNPs (homozyg‐window‐snp 100) and a minimum of ≥ 25 SNPs in a homozygous segment to be defined as a ROH (homozyg‐snp 25). The following parameters were set according to previous research (Hasselgren et al. 2021; Cockerill et al. 2022): a SNP must be within at least 5% of all windows to be defined as being within a homozygous segment (homozyg‐window‐threshold 0.05); and cover a total length of ≥ 100 kb (homozyg‐kb 100); minimum SNP density of one SNP per 50 kb (homozyg‐density 50) and the maximum distance between two neighbouring SNPs as ≤ 1000 kb (homozyg‐gap 1000). Heterozygous sites within a ROH at 750 (homozyg‐het 750) to prevent sequencing errors from fragmenting ROH. The autosomal inbreeding coefficient F_*ROH*_ was then estimated by summing the total length of ROH and dividing by the total length of the 25 longest scaffolds, giving the proportion of the genome in ROH.

To infer the timing of inbreeding events, we used the following equation (Kardos et al. 2018):

(1) $$g=100/\left(\text{2rL}\right)$$

Briefly, *g* corresponds to the number of generations, *L* is the length of ROH in Mb and *r* is the recombination rate (*r* = 4*NeC*; Hellmann et al. 2008), *C* denoting the probability of recombination. We used the recombination rate of the silver fox (0.6 cM per Mb; Kukekova et al. 2007) and the average generation time of 2 years for the arctic fox (Hasselgren et al. 2021; Cockerill et al. 2022). We evaluated the need to adjust generation time by ecotype, since coastal foxes and lemming foxes likely have shorter and longer generation times, respectively. However, to remain consistent with previous literature and keep comparative analyses within the same ROH lengths, we compromised with a 2‐year average generation time. To calculate F_*ROH*_ that corresponded to the generations prior‐to and following fur farming, we categorised the length of ROH by 133‐365 kb (∼600–300 generations back), 365–800 kb (∼300–100 generations back), 800–8 Mb (∼100–10 generations back) and > 8 Mb (∼10 generations back).

To test for significant differences between the ROH categories and between farmed populations and their respective wild populations for heterozygosity and F_*ROH*_, we used Kruskal‐Wallis and Dunn's tests for post hoc comparisons using the R package rstatix v.0.7.0 (Kassambara 2021).

## 3 Results

### 3.1 De‐Novo Assembly

We generated an arctic fox genome assembly using a combination of PacBio long‐read sequencing and Hi‐C scaffolding. The initial PacBio assembly produced a genome size of 2.56 Gb, comprising 3295 scaffolds with an N50 of 5.18 Mb. After Hi‐C scaffolding, contiguity improved significantly, resulting in a final assembly of 3444 scaffolds, with an N50 of 123.18 Mb. The majority of the genome is contained within 24 long scaffolds, likely representing chromosome‐scale structures. By examining synteny with the farmed arctic fox genome (Peng et al. 2021), we identified the X chromosome (HiC_scaffold_24).

### 3.2 Population Structure, Differentiation and Demographic History

**FIGURE 2:**
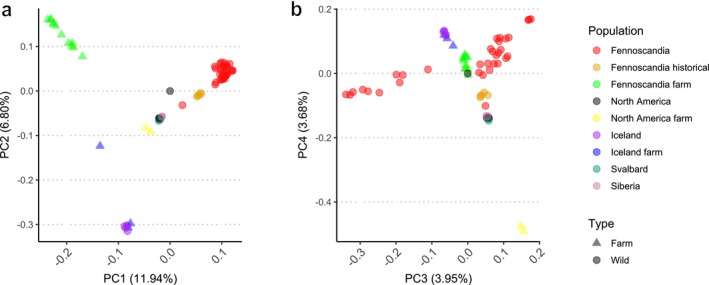
Population structure of farmed and wild arctic populations based on the genotype likelihoods. (a) Principal‐component analysis (PCA) showing population stratification on axes PC1‐PC2. (b) Principal‐component analysis (PCA) showing population stratification on axes PC3‐PC4.

**FIGURE 3:**
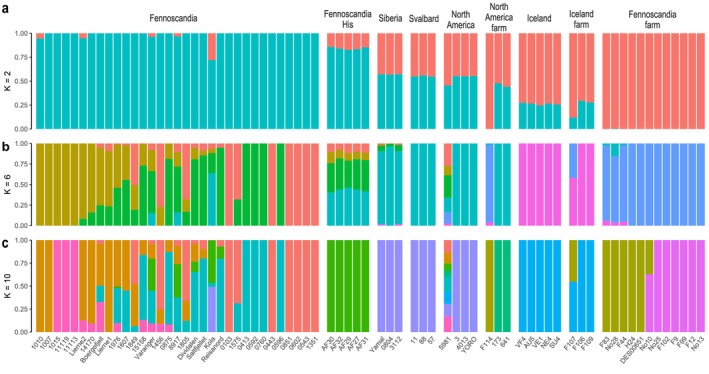
Admixture plot for inferred admixture proportions (farm: *N* = 18, wild: *N* = 53) at (a) *K* = 2, (b) *K* = 6 and (c) *K* = 10 based on genotype likelihoods.

We sequenced 43 genomes, achieving a final combined dataset of 71 arctic fox genomes from across their circumpolar range with an average depth of 16× (5.44–51×). The dataset represents 53 wild and 18 farmed individuals, covering key wild and farmed populations across the geographic distribution range. Using genotype likelihoods and SNP‐based analyses, we detected moderate population structure and differentiation between respective populations based on PCA and admixture analyses, as expected given geographic separation (Figures 2 and 3; Figure S2). The optimal K inferred by CLUMPAK was *K* = 2, but the residual correlations at *K* = 10 indicated unaccounted population substructure. This suggests that our data may have violated one or more model assumptions, such as unrelated individuals, Hardy–Weinberg equilibrium, absence of linkage disequilibrium, or the presence of unadmixed individuals representing each ancestral population without subsequent drift. Given the lack of confidence in the best *K*, we relied on the biological context and found that *K* = 6 separated the populations into known geographic locations. Firstly, *K* = 2 revealed distinct patterns between wild and farmed populations, suggesting considerable divergence. Fennoscandian farmed foxes and one North American farmed fox (F114) were identified as a single population with almost 100% assignment, whereas the Fennoscandian wild population had proportions assigned mostly to the other population. These patterns generally reflect pronounced divergence. In a similar pattern, Icelandic farmed foxes and wild foxes were assigned roughly (∼75%) of the second inferred population, however, one farmed fox was assigned closer to (∼80%). The samples from North America, Svalbard and Russia were more complex, however, and various proportions per individual may reflect ongoing gene flow or shared ancestry among the populations. Considering *K* = 6, we can see clear separation of population assignments based on geographic location, with Iceland, circumpolar foxes (North America, Siberia, Svalbard), Fennoscandian farmed foxes and two North American farmed foxes (173 and 641) forming discrete clusters. Fennoscandia is separated into two clusters, a north to south cline, with the more northern individuals (Varanger, 8917, Kola) showing signs of admixture with the circumpolar arctic fox population, indicating past connectivity to Siberia. One North American farmed fox (F114) clustered with Fennoscandian farmed foxes, and together with three Fennoscandian farmed foxes (F83, No28, F44), exhibit a small amount of admixture with wild Icelandic foxes (< 5%). Two of these foxes (F83, F44) also showed small levels of admixture with the circumpolar population at *K* = 6 (< 5%), while sample No28 showed greater admixture at roughly 20%. Among the putative Icelandic farmed foxes, one individual showed intermediate admixture proportions between Fennoscandian farmed foxes and Icelandic wild foxes (~50%), consistent at all *K*. At *K* = 6, the two farmed foxes from the Aleutian Islands (173 and 641) were assigned 100% to the circumpolar population, whereas they formed their own distinct cluster at K = 10. When considering K values, beyond K = 6, we begin to see additional clustering within Fennoscandia (Figure S3), reflecting the fragmented state of the population relative to other populations.

**FIGURE 4:**
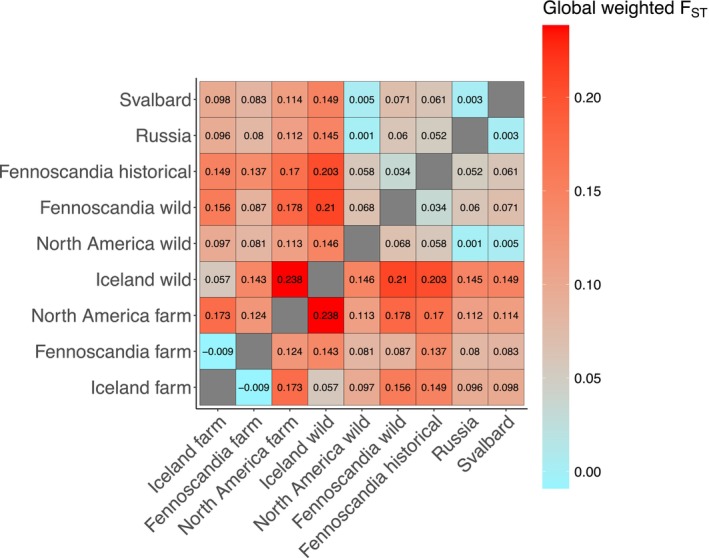
Genetic differentiation among farmed and wild arctic fox populations based on pairwise FST estimates calculated with ANGSD. Analyses were based on the derived SFS for all population pairings incorporating the weighted estimator from (Bhatia et al. 2013).

Global F_*ST*_ estimates ranged from −0.0009 to 0.238 (Figure 4). The lowest F_*ST*_ value among the comparison of farmed versus wild populations was found between the Icelandic farmed foxes and Icelandic wild foxes. In addition, the lowest F_*ST*_ values from Fennoscandian farmed foxes were seen in comparison with the wild populations of Russia, North America, Svalbard, and Fennoscandia. In contract, the highest F_*ST*_ value from the Fennoscandian farmed population was the Icelandic wild population.

**FIGURE 5:**
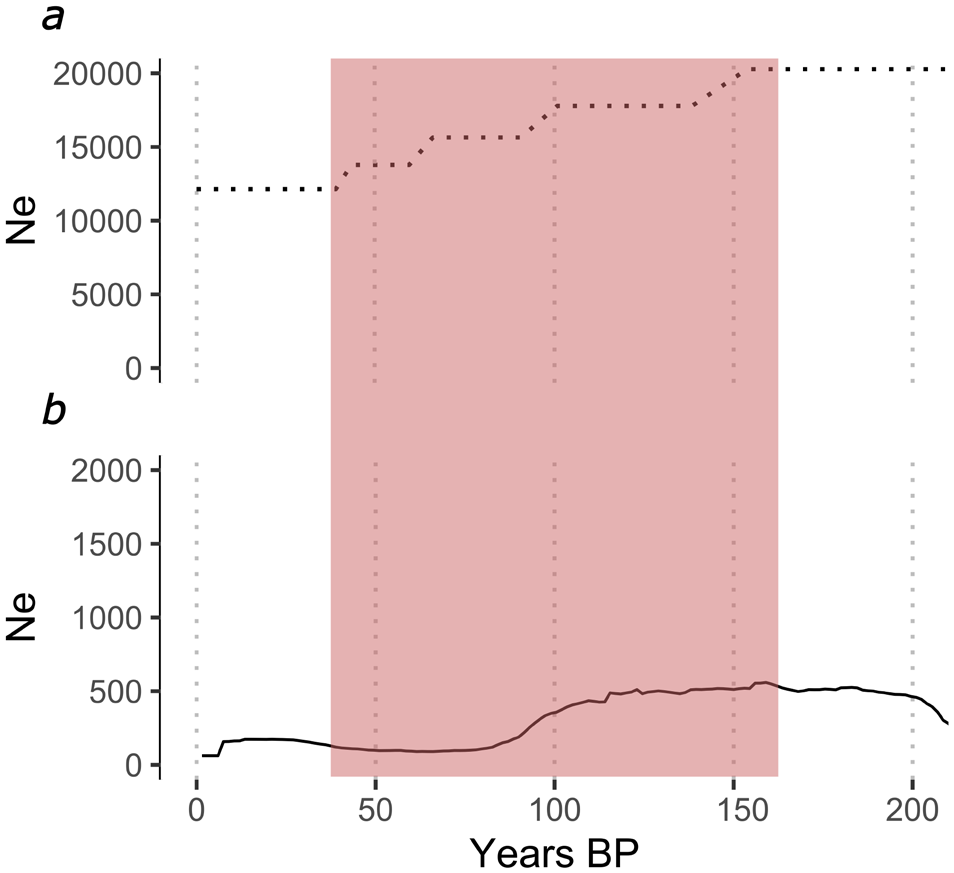
Recent demographic history reconstruction of the Fennoscandian farmed arctic fox population using effective population size (*Ne*). (a) The coalescence‐based inference implemented in SMC++. (b) The Linkage Disequilibrium‐based inference implemented in GoNe, showing (*Ne*) curves for the geometric mean over 40 independent runs. The *y*‐axis corresponds to (*Ne*) and the *x*‐axes are in years before present, scaled according to an average 1.5 year generation time. (*Ne*) estimates for SMC++ were roughly one order of magnitude higher than those inferred by GoNe. The vertical red bar represents the potential domestication bottleneck beginning ∼150 years BP.

SMC++ analysis detected an *Ne* decline beginning ∼350 years BP with subsequent declines at ∼240 years BP and ∼150 years BP and a more gradual decline until ∼35 years BP (Figure 5). GoNe detected a less severe, gradual decline beginning at ∼160 years BP with a steeper decline ∼110 years BP, followed by a gradual increase beginning ∼35 years BP (Figure 5). Both analyses detected a decline beginning at ∼150 years BP, represented by the red region. An increase in (*Ne*) was observed between ∼250 and 200 years BP in the GoNe analysis. The estimates of *Ne* from GoNe were 20 times lower than those of SMC++, reflecting differences in temporal resolution. GoNe is optimised for recent history (< 200 generations), capturing sharp declines through linkage disequilibrium. In contrast, SMC++ employs a coalescent‐based approach that smooths over recent changes, yielding higher estimates reflecting broader, long‐term trends. In this case, the *Ne* estimates from GoNe provide a more accurate reflection of recent population dynamics, while the key insight from SMC++ is its consistent timing of decline with GoNe.

### 3.3 Phylogenetic Analyses

**FIGURE 6:**
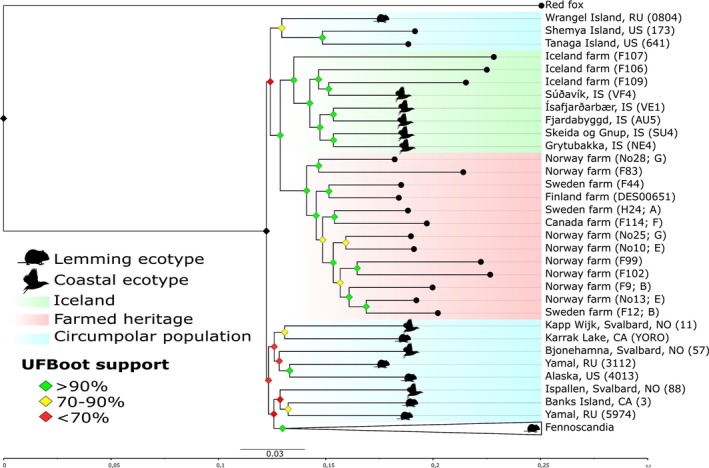
Maximum Likelihood phylogeny constructed using BAM files (farm: *N* = 18, wild: *N* = 53). Blue indicates the circumpolar arctic fox, red represents the farmed fox clade, green represents Icelandic wild and farmed foxes. We collapsed the Fennoscandian clade for illustrative purposes. Diamonds at the nodes indicate Ultra Fast Bootstrap support; Red ≤ 70%, Yellow = 70%–90% and Green > 90%. Sample ID followed by breeding line for each sample are in parentheses.

The maximum likelihood phylogeny placed the putative Icelandic farmed foxes within the Icelandic clade, which was sister to a Fennoscandian farmed fox clade, each with 100% bootstrap support (Figure 6). There were two major clades identified; however, they had relatively low bootstrap support resulting in many alternative topologies. One major clade placed the Shemya and Tanaga Island samples as sisters with high support, with Wrangel Island diverging earlier with moderate support (70%–90%). This clade was assigned sisters with a clade consisting of wild Icelandic foxes with low support. Consistent with the structure analyses, the putative Icelandic farmed fox sample F107 diverged earlier than the rest of the wild Icelandic foxes and the remaining Icelandic foxes formed two inner sister clades: a clade consisting of a wild individual from Súðavík, north‐west Iceland with the putative farmed foxes F106 and F109, and a clade consisting of only Icelandic wild foxes. All three putative Icelandic farmed foxes had longer branch lengths than wild foxes, indicating greater divergence relative to the overall phylogeny, measured as substitutions per site. The same pattern was also seen in three Fennoscandian farmed fox individuals and within the Fennoscandian wild clade.

Only wild foxes were assigned to the second major clade, which had lower bootstrap support at the inner nodes than the first major clade. Only the inner branches of this clade had high support; Yamal with Alaska and the entire Fennoscandian wild population had 100% bootstrap support, while Kapp Wijk, Svalbard, NO (11) with Karrak Lake, CA (YORO) and Banks Island with Yamal, RU (5974) had medium support.

### 3.4 Heterozygosity and Inbreeding

**FIGURE 7:**
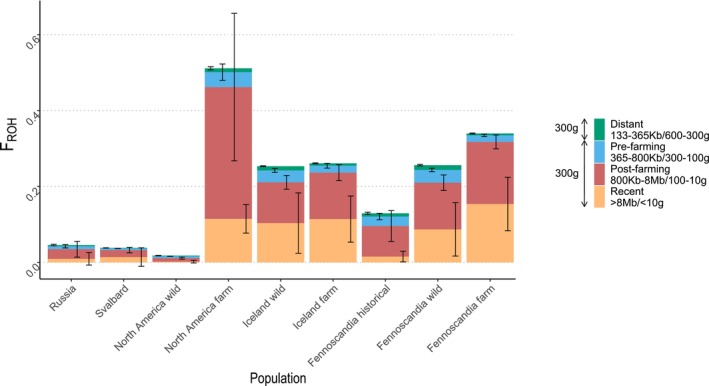
Mean genomic inbreeding coefficients (*F*_ROH_) with standard deviation for wild and farmed arctic fox populations. Green bars show inbreeding due to common ancestors in the distant past (300–600 generations, 133–365 kb), blue bars show inbreeding due to common ancestors 300–100 generations back (365–800 kb), red bars show inbreeding due to common ancestors 100–10 generations back (800 kb—8 Mb) and yellow bars show inbreeding due to recent common ancestors less than 10 generations back (> 8Mb).

Overall, both wild and farmed populations exhibited a similarly low average proportion of their genomes in ROH, reflecting inbreeding from common ancestors in the distant past (133–365 kb; 300–600 generations; Figure 7). This proportion showed a slight increase across all populations when considering ROH lengths corresponding to the pre‐farming period (365–800 kb; 100–300 generations). In contrast, a substantial rise was observed during the post‐farming period, indicating more recent inbreeding, followed by a notable decrease in ROH lengths associated with the past 10 generations (> 8 Mb; < 10 generations). However, the extent of this latter reduction was highly population‐dependent. Notably, Iceland and Fennoscandia remained at a high proportion of ROH in the past 10 generations relative to the other wild populations. Among the farmed arctic fox populations, Fennoscandia exhibited a significantly higher average proportion of the genome in ROH compared to their wild counterparts in Fennoscandia, however demonstrated 83% lower average inbreeding between common ancestors in the distant past (ROH 133‐365 kb; 300–600 generations; *χ* ^2^ = 50.192; *p* = < 0.001), 61% lower inbreeding prior to farm establishment (365–800 kb; 100–300 generations; *χ* ^2^ = 45.788; *p* = 0.001), and 41% higher in the post‐farming period (ROH 800 kb‐8 Mb; 100–10 generations; *χ* ^2^ = 35.408; *p* = 0.00773). There was, however, no significant difference for inbreeding between common ancestors during the last 10 generations (ROH 800 kb‐8 Mb, 100–10 generations). North American farmed foxes demonstrated the largest proportion of the genome in ROH, the majority due to inbreeding between common ancestors in the post‐farming category, showing an increase of 195% from the North American wild population (ROH 800 kb‐8 Mb, 100–10 generations; *χ* ^2^ = 48.622; *p* = 0.013). However, there was no significant difference for the other ROH categories.

**FIGURE 8:**
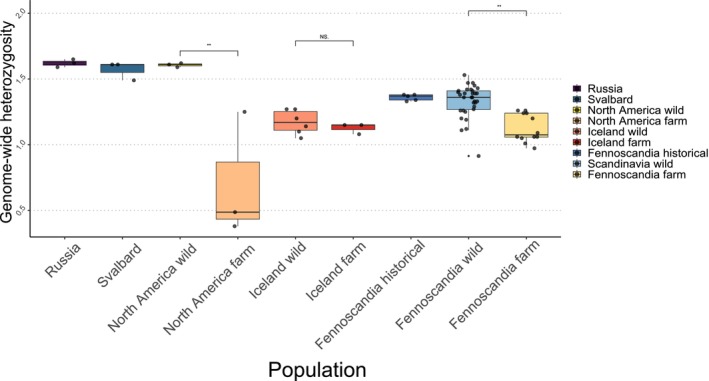
Genome‐wide heterozygosity as heterozygous sites per 1 kb. The horizontal bar shows the median, the boxes show the 25%–75% inter‐quartile range, the whiskers show the whole range. Significant results marked with *p < 0.05, **p < 0.01, ***p < 0.001.

Genome‐wide heterozygosity exhibited similar trends with increased heterozygosity in the circumpolar populations, whereas Iceland wild, Iceland farm, Fennoscandia wild, farm and historical populations showed lower levels of heterozygosity (Figure 8). Consistent with the ROH results, the Fennoscandian farm population exhibited a significant reduction in genome‐wide heterozygosity levels compared to the wild population of Fennoscandia (*χ* ^2^ = 46.364; *p* = 0.00805). Moreover, the North American farm population had significantly lower genome‐wide heterozygosity than the North American wild population (*χ* ^2^ = 46.364; *p* = 0.01928).

## 4 Discussion

We investigated the demographic history of semi‐domesticated, farmed arctic fox populations by detecting the genomic consequences of intensive fur farming using genome data from 71 wild and farmed arctic foxes across the species' circumpolar distribution. Overall, our results highlight the direct genomic consequences of captive breeding in arctic foxes, namely, founder effects, chronically low effective population sizes, and increased inbreeding due to mating among close relatives. These findings underscore how historical fur farming practices, driven primarily by production priorities rather than animal welfare, have left a lasting imprint on the genome.

The close assignment between Fennoscandian farmed foxes and the isolated, coastal Icelandic wild population is consistent with historical records of Icelandic arctic fox ancestry (Nes et al. 1987). This result suggests that introgression from Fennoscandian farm foxes into the wild Fennoscandian population could increase the risk of losing genes adapted for rodent cycles. Furthermore, the observed admixture between Icelandic and Fennoscandian farmed foxes indicates movement between wild Icelandic foxes and the farmed populations, either in situ or ex situ. This is consistent with historical accounts of farmed foxes being imported from Norway to Iceland in two waves (1930–1950s and 1980–1990s; Unnsteinsdottir, pers. comm).

Importantly, our analyses reveal that the genomic erosion observed in farmed foxes is not solely a signature of past intensive breeding but also reflects the dynamic interplay between domesticated and wild congeners. When captive‐bred individuals come into contact with wild populations, multiple outcomes may occur, including admixture with subsequent negative or beneficial fitness effects (i.e., outbreeding depression versus genetic rescue) and the potential for “re‐wilding” of areas with populations pre‐adapted to low population sizes. In the case of Tanaga and Shemya Islands, feral populations established from captive stock persist today, suggesting that domestication may, under certain circumstances, be partially reversible. This observation feeds into the broader, and increasingly debated, concept of naturalisation, where feral and hybrid populations might exhibit similar, or even enhanced viability within vacant or shifting niche spaces.

Our results also demonstrate that, while genomics offers a powerful tool to elucidate demographic history and the consequences of artificial selection, relying solely on a single method can be problematic. The convergence of evidence from SMC++, GoNe, and ROH analyses provides a robust signal of a domestication bottleneck approximately 150 years BP, despite inherent uncertainties (e.g., in the genetic mutation rate) common in non‐model organisms. Such cross‐validation is crucial for interpreting the complex processes of genetic drift, inbreeding, and founder effects in both domesticated and re‐wilding contexts. Similar signatures of inbreeding reflecting a domestication bottleneck have been detected in the American mink ( *Neovison vison* ) genome (Guldbrandtsen et al. 2018).

Fur farming practices have historically faced public scrutiny, particularly regarding animal welfare and breeding strategies. Although laws have been implemented to maintain adequate care of captive animals, the consequences of former breeding practices can have long‐lasting impacts. The underlying genomic composition that underpins individual fitness and health must be considered, especially given that attempts to minimize illness and disease in arctic fox fur farms (Kempe and Strandén 2016) have been largely driven by economic production priorities. While there has been a trend toward the abolition of fur farms in the past two decades, many farms persist. Our results demonstrate that the consequences of fur farming are not always visible. Although livestock genomics has begun to implement genomic insights into breeding practices and animal management to prevent negative outcomes (Windig et al. 2004; Windig and Doekes 2018; Doekes et al. 2018; Charlier et al. 2008, 2016; Derks et al. 2019), many species, especially those within the fur industry, remain neglected.

In conclusion, our study not only documents the genomic impact of fur farming in arctic foxes, but also raises broader questions about managing feral and hybrid populations. As natural habitats change and niches vacate, understanding the balance between genome erosion in captive and wild populations and the implications of domestic lineages in the wild will be critical. Further research, particularly into the mutational load and fitness consequences of these breeding practices, will help clarify the long‐term viability of both captive and vulnerable wild populations, as well as the potential for “re‐wilded” populations in the context of a rapidly changing environment.

## Author Contributions

C.A.C. and K.N. designed and supervised the study. C.A.C., N.B., J.S., G.B., M.H., J.W., A.A., E.F., E.R.U., P.W., G.S., R.A., D.B., Ø.F., A.L., N.E.E., L.D. and K.N. prepared the samples. C.A.C. and J.C.C.‐D. were involved in the data analyses. R.‐A.O. and I.B. generated the de novo assembly. S.P. and K.P.M. provided resources for the de novo assembly. C.A.C., J.C.C.‐D., J.S., L.D. and K.N. discussed and interpreted the results. C.A.C. wrote the manuscript with the other authors' assistance. All authors revised the draft and approved the final manuscript.

## Disclosure

*Benefit‐Sharing*: This research was conducted in collaboration with researchers from institutions in the countries of origin of the samples, and all contributors are included as coauthors. The genomic data generated have been archived in publicly accessible repositories for use by the wider scientific community. The research directly addresses conservation concerns by providing insights into the genomic consequences of fur farming and the potential risks of introgression into wild arctic fox populations, information that can support future monitoring and management strategies. More broadly, this work contributes to international research partnerships and increases publicly available genomic resources for Arctic fauna.

## Conflicts of Interest

The authors declare no conflicts of interest.

## Supporting information


**Data S1:** mec70166‐sup‐0001‐Supinfo.pdf.

## Data Availability

The De‐Novo assembly has been deposited to the European Nucleotide Archive (ENA) under project PRJEB71153 with the accession number GCA_963924105. The whole‐genome sequencing data generated for this study are available to download at ENA under the accession number PRJEB97417. Sequencing data downloaded for this study from Hasselgren et al. (2021), Cockerill et al. (2022) and von Seth et al. (2025) are available to download at ENA under accession numbers PRJEB55788, PRJEB43377 and PRJEB77095, respectively. Illumina reads from Peng et al. (2021) are available at the NCBI database under the accession number PRJNA597264.
