# Supplementary material for: That's So Last Season: Unraveling the Genomic Consequences of Fur Farming in Arctic Foxes (Vulpes lagopus)
Source: Mol Ecol. 2025 Nov 13;34(24):e70166. doi: 10.1111/mec.70166 (PMC12717966; doi:10.1111/mec.70166)
Supplement: Supplementary file 1 — Data S1: mec70166‐sup‐0001‐Supinfo.pdf. [file MEC-34-e70166-s001.pdf]

MOLECULAR ECOLOGY

Supplemental Information for:

That's so Last Season: Unraveling the Genomic Consequences of Fur Farming in Arctic Foxes (*Vulpes lagopus*)

Christopher A. Cockerill, Camilo Chacón-Duque, Nora Bergfeldt, Joanna von Seth, Gabriella Björklund, Malin Hasselgren, Johan Wallén, Anders Angerbjörn, Eva Fuglei, Ester Rut Unnsteinsdottir, Paula White, Gustaf Samelius, Ray Alisauskas, Dominique Berteaux, Øystein Flagstad, Arild Landa, Nina E. Eide, Love Dalén and Karin Norén

Table of Contents:

**Table S1**	Page 2
**Table S1**	Page 3
**Table S1**	Page 4
**Table S1, Table S2, Table S3**	Page 5
**Table S4, Table S5**	Page 6
**Table S6, Figure S1**	Page 7
**Figure S2**	Page 8
**Figure S3**	Page 9
**Figure S3**	Page 10

MOLECULAR ECOLOGY

Table S1: Genome-based individual genetic variation in the sample areas of Russia, Svalbard, North America, Iceland and Fennoscandia. Sequencing coverage; Het/kb = heterozygous sites per 1000 bp; F_{ROH} > 133-365kb = proportion of genome contained within ROH between 133 and 365kb long; F_{ROH} >365-800kb = proportion of genome contained in ROH between 365-800kb long; FROH > 800kb-8Mb = proportion of genome contained in ROH between 800kb and 8Mb long.

FID	133-365kb	365-800kb	800kb-8Mb	>8Mb	Het/kb	Coverage	Sequencing instrument
3	0,001137	0,005103	0,011905	0	1,62	9,87	Illumina TruSeq PCR-free Covaris 350bp
11	0,001286	0,003828	0,012736	0	1,61	13,25	Illumina TruSeq PCR-free Covaris 350bp
57	0,001999	0,004598	0,026503	0,042231	1,49	10,94	Illumina TruSeq PCR-free Covaris 350bp
88	0,001116	0,004177	0,016124	0	1,61	15,67	Illumina TruSeq PCR-free Covaris 350bp
103	0,011796	0,030632	0,092087	0,092136	1,39	16,51	Illumina TruSeq PCR-free 350bp
173	0,01109	0,050002	0,534023	0,157599	0,38	14,99	Illumina TruSeq PCR-free Covaris 350bp
413	0,011892	0,033435	0,115325	0,072997	1,39	14,43	Illumina TruSeq PCR-free 350bp
443	0,012367	0,033133	0,107907	0,195043	1,19	12,82	Illumina TruSeq PCR-free 350bp
543	0,010904	0,034462	0,119006	0,130646	1,27	17,10	Illumina TruSeq PCR-free 350bp
592	0,013726	0,039276	0,114114	0,039029	1,45	17,79	Illumina TruSeq PCR-free 350bp
596	0,012366	0,037165	0,126322	0,033431	1.44	27,17	Illumina TruSeq PCR-free 350bp
602	0,011051	0,031479	0,107415	0,135223	1,26	16,43	Illumina TruSeq PCR-free 350bp
641	0,01384	0,053529	0,361251	0,0875	0,00	15,84	Illumina TruSeq PCR-free Covaris 350bp
760	0,008839	0,026394	0,102021	0,248152	1,12	19,14	Illumina TruSeq PCR-free 350bp
804	0,002635	0,004763	0,018598	0	1,62	31,78	Illumina TruSeq PCR-free 350bp
851	0,012094	0,028015	0,101355	0,212914	1,20	16,95	Illumina TruSeq PCR-free 350bp
875	0,009938	0,029582	0,132153	0,235673	1,11	22,49	Illumina TruSeq PCR-free 350bp
1007	0,014253	0,037933	0,114595	0,051184	1,41	15,86	Illumina TruSeq PCR-free 350bp

MOLECULAR ECOLOGY

1010	0,014129	0,03343	0,093616	0,046443	1,42	16,99	Illumina TruSeq PCR-free 350bp
1015	0,014534	0,035581	0,143475	0,063075	1,32	16,36	Illumina TruSeq PCR-free 350bp
1351	0,007496	0,02202	0,087308	0,373816	0,91	17,35	Illumina TruSeq PCR-free 350bp
1456	0,011867	0,033423	0,150173	0,087436	1,29	17,00	Illumina TruSeq PCR-free 350bp
1575	0,01293	0,040208	0,149333	0,054513	1,36	19,76	Illumina TruSeq PCR-free 350bp
1607	0,014043	0,032878	0,118218	0,09082	1,39	19,37	Illumina TruSeq PCR-free 350bp
1805	0,01232	0,038578	0,109426	0,104571	1,38	16,68	Illumina TruSeq PCR-free 350bp
1849	0,014792	0,034482	0,133309	0,10847	1,33	21,47	Illumina TruSeq PCR-free 350bp
1976	0,012561	0,031673	0,134837	0,100495	1,32	21,74	Illumina TruSeq PCR-free 350bp
3112	0,001711	0,005827	0,007811	0	1,65	17,43	Illumina TruSeq PCR-free 350bp
4013	0,002462	0,00467	0,006619	0	1,61	17,80	Illumina TruSeq PCR-free Covaris 350bp
8917	0,00967	0,028965	0,147043	0,053321	1,39	26,57	Illumina TruSeq PCR-free 350bp
11113	0,010821	0,035699	0,113159	0,148867	1,26	16,43	Illumina TruSeq PCR-free 350bp
11119	0,012046	0,033667	0,14814	0,095465	1,25	18,90	Lucigen NxSeq AmpFREE Low 350bp
14170	0,015091	0,037567	0,103916	0,085281	1,41	24,82	Illumina TruSeq PCR-free 350bp
15158	0,013951	0,038776	0,157947	0,071356	1,36	22,82	Illumina TruSeq PCR-free 350bp
AF27	0,009824	0,027582	0,081524	0,02795	1.38	8.27541	Meyer & Kircher (2010) Illumina HiSeqX Ten
AF29	0,011868	0,032613	0,101132	0,031784	1,34	13.2852	Meyer & Kircher (2010) Illumina HiSeqX Ten
AF30	0,004583	0,010942	0,01295	0	1,33	10.1966	Meyer & Kircher (2010) Illumina HiSeqX Ten
AF31	0,008615	0,029725	0,086697	0,013471	1,38	14.0134	Meyer & Kircher (2010) Illumina HiSeqX Ten
AF32	0,007692	0,023786	0,120165	0,004081	1,37	11.3058	Meyer & Kircher (2010) Illumina HiSeqX Ten
AU5	0,013294	0,036856	0,104173	0,012476	1,27	17.8218	Illumina TruSeq PCR-free Covaris 350bp
Boergefjell	0,013799	0,031729	0,13082	0,068974	1,36	19,57	-
DES00651	0,00821	0,025338	0,165756	0,05356	1,20	51,51	-

Dividalen	0,010723	0,032583	0,122055	0,060442	1,40	13,93	-
F102	0,005941	0,018676	0,188818	0,218094	1,01	11,53	Illumina TruSeq PCR-free Covaris 350bp
F106	0,00681	0,019969	0,145141	0,049292	1,15	11,85	Illumina TruSeq PCR-free Covaris 350bp
F107	0,00494	0,011315	0,117856	0,123446	1,15	14,37	Illumina TruSeq PCR-free Covaris 350bp
F109	0,007311	0,023482	0,103872	0,169807	1,08	13,75	Illumina TruSeq PCR-free Covaris 350bp
F114	0,005571	0,014518	0,14608	0,099353	1,25	12,81	Illumina TruSeq PCR-free Covaris 350bp
F12	0,004918	0,016739	0,154156	0,196561	1,06	13,36	Illumina TruSeq PCR-free Covaris 350bp
F44	0,005371	0,014427	0,17691	0,070671	1.24	14,20	Illumina TruSeq PCR-free 350bp
F83	0,004911	0,019328	0,149272	0,085075	1,24	13,17	Illumina TruSeq PCR-free Covaris 350bp
F9	0,004037	0,017092	0,175386	0,184395	1,06	9,92	Illumina TruSeq PCR-free Covaris 350bp
F99	0,004991	0,015011	0,195538	0,258221	0,97	11,92	Illumina TruSeq PCR-free Covaris 350bp
H24	0,003467	0,015199	0,148601	0,107652	1,26	16,03	Illumina TruSeq PCR-free 350bp
Kola	0,00533	0,01303	0,048249	0,028526	1,53	7,51	-
Lierne1	0,01425	0,034264	0,110771	0,034067	1,47	16,15	-
Lierne2	0,011005	0,032535	0,126015	0,091446	1,33	13,81	-
NE4	0,010305	0,024226	0,093483	0,200328	1,05	12,53	Illumina TruSeq PCR-free Covaris 350bp
No10	0,003756	0,016473	0,162109	0,168266	1,09	14,71	Illumina TruSeq PCR-free 350bp
No13	0,004505	0,013849	0,161179	0,21714	1,06	12,74	Illumina TruSeq PCR-free 350bp
No25	0,004034	0,015523	0,153302	0,209595	1,05	18,34	Illumina TruSeq PCR-free 350bp
No28	0,0053	0,020256	0,131058	0,078168	1,26	16,42	Illumina TruSeq PCR-free 350bp
Reisanord	0,013418	0,037403	0,109815	0,033829	1,42	14,73	-
Saltfjellet	0,011386	0,033702	0,102765	0,027983	1,47	7,85	-
SU4	0,010419	0,03015	0,097743	0,165803	1,10	16,98	Illumina TruSeq PCR-free Covaris 350bp
Varanger	0,01162	0,023611	0,098539	0,063348	1,43	17,90	-

MOLECULAR ECOLOGY

VE1	0,011124	0,029604	0,138635	0,096797	1,14	17,57	Illumina TruSeq PCR-free Covaris 350bp
VF4	0,011611	0,035424	0,102807	0,042596	1,27	15,60	Illumina TruSeq PCR-free Covaris 350bp
Yamal	0,002241	0,004705	0,019864	0	1,59	6,94	-
YORO	0,001877	0,004584	0,009761	0,005773	1,59	12,57	Illumina TruSeq PCR-free Covaris 350bp

Table S2: Results of the post hoc Dunn's tests showing significance of difference of genome-wide heterozygosity among Russia, Svalbard, North America wild, North America farm, Iceland wild, Iceland farm, Fennoscandia historical wild, Fennoscandia modern wild and Fennoscandia modern farm. Significant values in bold with * P<0.05, ** P<0.01, ***P<0.001.

F_{ROH}	Russia	Svalbard	NA wild	NA farm	IS wild	IS farm	Fenno his	Fenno wild	Fenno farm
**Russia**	-	-	-	-	-	-	-	-	-
**Svalbard**	NS	-	-	-	-	-	-	-	-
**NA wild**	NS	NS	-	-	-	-	-	-	-
**NA farm**	0.00813 **	0.02596 *	0.01928 *	-	-	-	-	-	-
**IS wild**	0.01965 *	NS	0.04716 *	NS	-	-	-	-	-
**IS farm**	0.03106 *	NS	NS	NS	-	-	-	-	-
**Fenno his**	NS	NS	NS	NS	NS	NS	-	-	-
**Fenno wild**	NS	NS	NS	NS	NS	NS	NS	-	-
**Fenno farm**	0.00043 ***	0.00514 **	0.00315 **	NS	NS	NS	NS	0.00805 **	-

Table S3: Results of the post hoc Dunn's tests, showing significance of difference in inbreeding due to common ancestors 300-600 generations back among Russia, Svalbard, North America wild, North America farm, Iceland wild, Iceland farm, Fennoscandia historical wild, Fennoscandia modern wild and Fennoscandia modern farm. Significant values in bold with * P<0.05, ** P<0.01, ***P<0.001.

F_{ROH}	Russia	Svalbard	NA wild	NA farm	IS wild	IS farm	Fenno his	Fenno wild	Fenno farm
**Russia**	-								
**Svalbard**	NS	-							
**NA wild**	NS	NS	-						
**NA farm**	NS	NS	NS	-					
**IS wild**	NS	NS	NS	NS	-				
**IS farm**	NS	NS	NS	NS	NS	-			
**Fenno his**	NS	NS	NS	NS	NS	NS	-		

**Fenno wild**	**0.0119 ***	**0.0043 ****	**0.0072 ****	NS	NS	NS	NS	-	
**Fenno farm**	NS	NS	NS	NS	NS	NS	NS	**4.7e-05 *****	-

Table S4: Results of the post hoc Dunn's tests, showing significance of difference in inbreeding due to common ancestors 100-300 generations back among Russia, Svalbard, North America wild, North America farm, Iceland wild, Iceland farm, Fennoscandia historical wild, Fennoscandia modern wild and Fennoscandia modern farm. Significant values in bold with * P<0.05, ** P<0.01, ***P<0.001.

F_{ROH}	Russia	Svalbard	NA wild	NA farm	IS wild	IS farm	Fenno his	Fenno wild	Fenno farm
**Russia**	-								
**Svalbard**	NS	-							
**NA wild**	NS	NS	-						
**NA farm**	NS	NS	NS	-					
**IS wild**	NS	NS	NS	NS	-				
**IS farm**	NS	NS	NS	NS	NS	-			
**Fenno his**	NS	NS	NS	NS	NS	NS	-		
**Fenno wild**	**0.0250 ***	NS	**0.0141 ***	NS	NS	NS	NS	-	
**Fenno farm**	NS	NS	NS	NS	NS	NS	NS	**0.0010 ****	-

Table S5: Results of the post hoc Dunn's tests showing significance of difference in inbreeding due to common ancestors 10-100 generations back among Russia, Svalbard, North America wild, North America farm, Iceland wild, Iceland farm, Fennoscandia historical wild, Fennoscandia modern wild and Fennoscandia modern farm. Significant values in bold with * P<0.05, ** P<0.01, ***P<0.001.

F_{ROH}	Russia	Svalbard	NA wild	NA farm	IS wild	IS farm	Fenno his	Fenno wild	Fenno farm
**Russia**	-								
**Svalbard**	NS	-							
**NA wild**	NS	NS	-						
**NA farm**	**0.01679 ***	**0.02026 ***	**0.00773 ****	-					
**IS wild**	NS	NS	NS	NS	-				
**IS farm**	NS	NS	NS	NS	NS	-			
**Fenno his**	NS	NS	NS	NS	NS	NS	-		
**Fenno wild**	NS	NS	NS	NS	NS	NS	NS	-	
**Fenno farm**	**0.00117 ****	**0.00158 ****	**0.00034 *****	NS	**0.04703 ***	NS	**0.00274 ****	**0.00773 ****	-

MOLECULAR ECOLOGY

Table S6: Results of the post hoc Dunn's tests showing significance of difference in inbreeding due to common ancestors 10 generations ago among Russia, Svalbard, North America wild, North America farm, Iceland wild, Iceland farm, Fennoscandia historical wild, Fennoscandia modern wild and Fennoscandia modern farm. Significant values in bold with * P<0.05, ** P<0.01, ***P<0.001.

F_{ROH}	Russia	Svalbard	NA wild	NA farm	IS wild	IS farm	Fenno his	Fenno wild	Fenno farm
**Russia**	-								
**Svalbard**	NS	-							
**NA wild**	NS	NS	-						
**NA farm**	NS	NS	NS	-					
**IS wild**	NS	NS	NS	NS	-				
**IS farm**	NS	NS	NS	NS	NS	-			
**Fenno his**	NS	NS	NS	NS	NS	NS	-		
**Fenno wild**	NS	NS	NS	NS	NS	NS		-	
**Fenno farm**	**0.0109 ***	**0.0475 ***	**0.0180 ***	NS	NS	NS	**0.0056 ****	NS	-

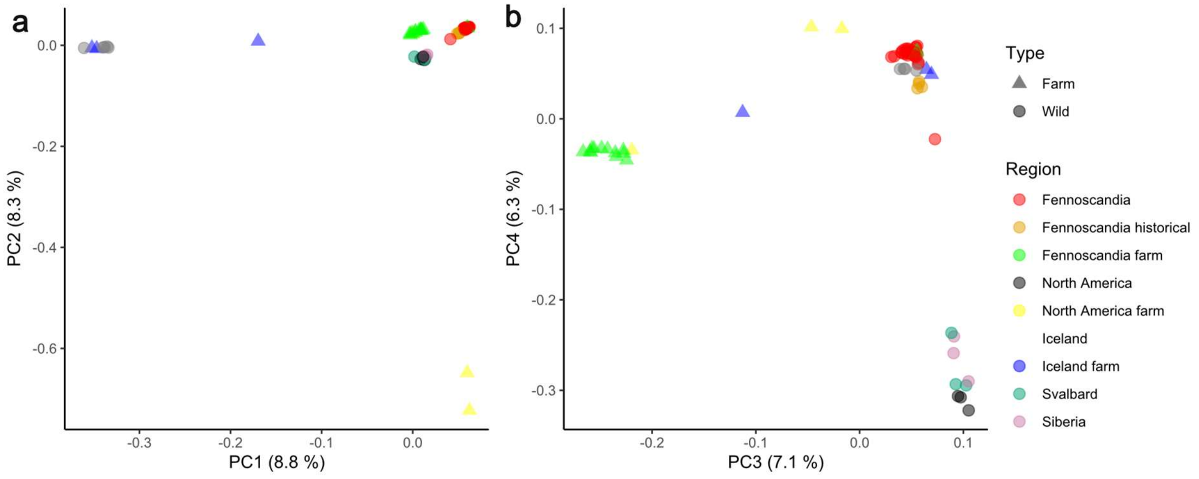

Figure S1 *Principal Component (PC) analyses based on Single Nucleotide Polymorphism data on axes a) PC1-PC2 and b) PC3-PC4*

MOLECULAR ECOLOGY

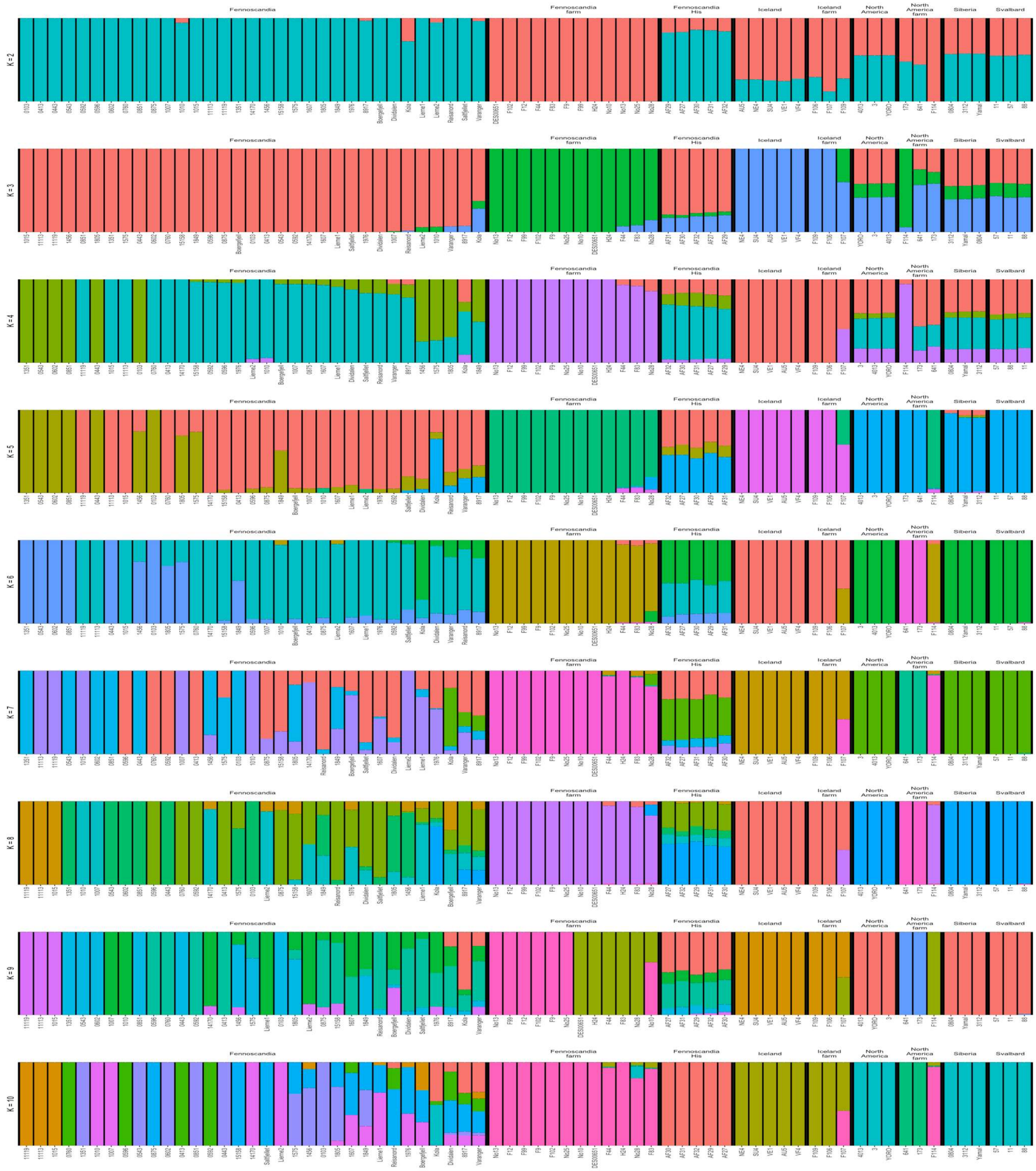

Figure S2 *Admixture plot for inferred admixture proportions (farm: n=18, wild: n=52) at K = 2 - K = 10 based on genotype likelihoods.*

MOLECULAR ECOLOGY

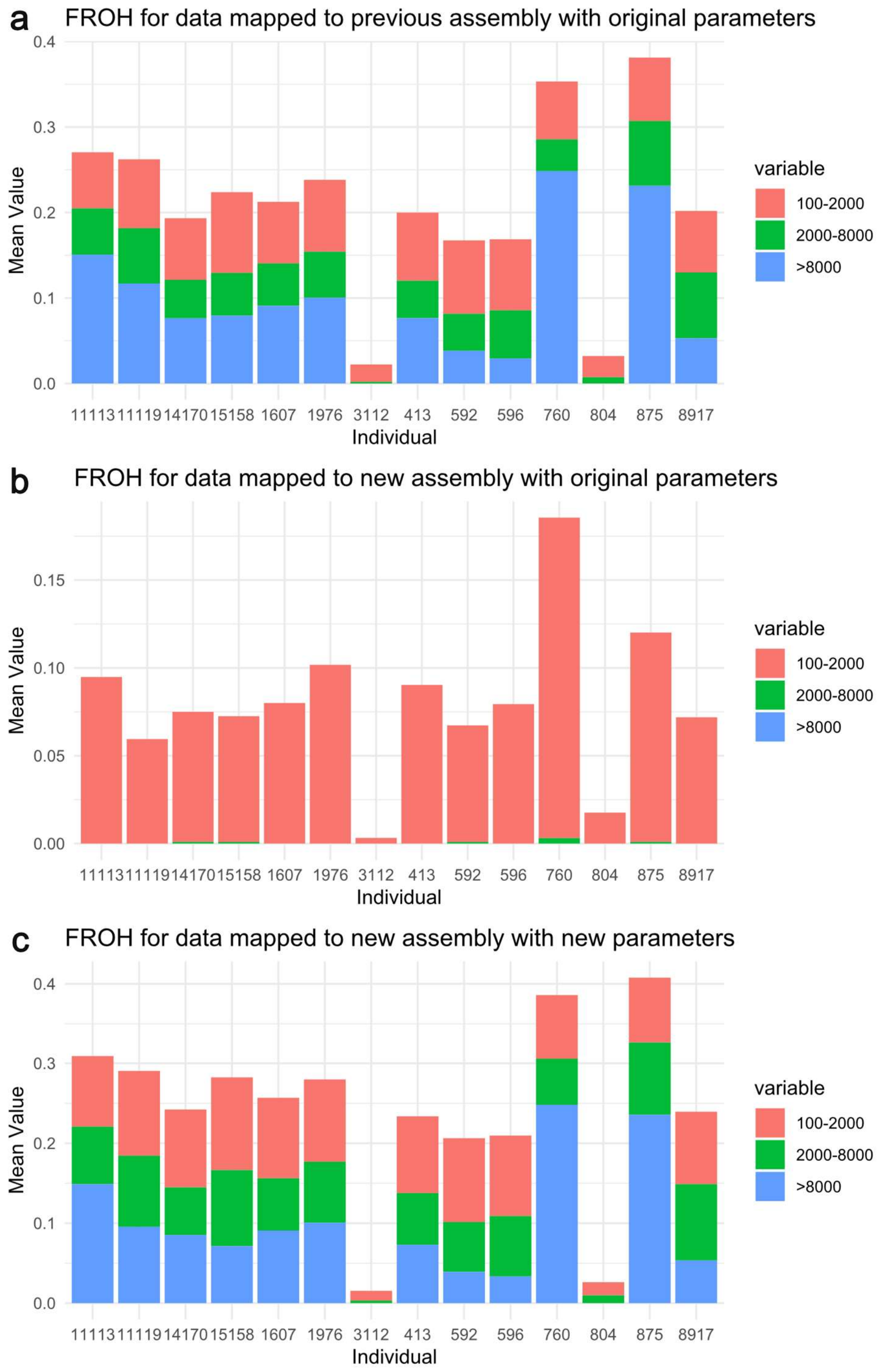

Figure S3 *Mean genomic inbreeding coefficients (F_{ROH}) with standard deviation for wild and farmed arctic fox populations. Red bars show inbreeding due to common ancestors in the distant past 45-850 (100-2 Mb), green bars show inbreeding due to common ancestors 10-45 generations back (2 Mb- 8 Mb) and blue bars show inbreeding*

MOLECULAR ECOLOGY

due to recent common ancestors less than 10 generations back (>8 Mb). a) shows inbreeding coefficients as seen in Cockerill et al., 2022 using a previous genome assembly and the study parameters, b) shows inbreeding coefficients from data mapped to the new reference genome (PRJEB71153) using the study parameters and c) inbreeding coefficients from data mapped to the new genome assembly with adjusted parameters.
